# Species-specific PCR to describe local-scale distributions of four cryptic species in the *Penicillium chrysogenum* complex^☆^

**DOI:** 10.1016/j.funeco.2013.04.003

**Published:** 2013-10

**Authors:** Alexander G.P. Browne, Matthew C. Fisher, Daniel A. Henk

**Affiliations:** Department of Infectious Disease Epidemiology, Imperial College London, London, United Kingdom

**Keywords:** Alexander Fleming, London Underground, Mycology, *Penicillium chrysogenum*, Phylogeny, Taxonomy

## Abstract

*Penicillium chrysogenum* is a ubiquitous airborne fungus detected in every sampled region of the Earth. Owing to its role in Alexander Fleming's serendipitous discovery of Penicillin in 1928, the fungus has generated widespread scientific interest; however its natural history is not well understood. Research has demonstrated speciation within *P*. *chrysogenum*, describing the existence of four cryptic species. To discriminate the four species, we developed protocols for species-specific diagnostic PCR directly from fungal conidia. 430 *Penicillium* isolates were collected to apply our rapid diagnostic tool and explore the distribution of these fungi across the London Underground rail transport system revealing significant differences between Underground lines. Phylogenetic analysis of multiple type isolates confirms that the ‘Fleming species’ should be named *Penicillium rubens* and that divergence of the four ‘Chrysogenum complex’ fungi occurred about 0.75 million yr ago. Finally, the formal naming of two new species, *Penicillium floreyi* and *Penicillium chainii*, is performed.

## Highlights

• We develop species-specific diagnostic PCR tools for four cryptic species.
• We apply our diagnostic tools in the London Underground transport system.
• Varied distributions of *Penicillium chrysogenum* are demonstrated.
• Alexander Fleming's fungus is confirmed as *Penicillium rubens.*
• Two new species in the ‘Chrysogenum complex’ are named: *P. floreyi* and *Penicillium chainii*.

## Introduction

*Penicillium chrysogenum* is one of the most numerous eukaryotes on earth with conidia present in concentrations up to 350 m^−3^ air and 15000 g^−1^ household dust (Beguin & Nolard 1994; Scott 2001). Even sampling from the upper-atmosphere and the Antarctic has detected this ubiquitous fungus (Pitt 2000), and as experienced by Alexander Fleming (Fleming 1929), avoiding *P. chrysogenum* as a contaminant is near impossible. Apart from readily acting as a food spoilage agent (Samson *et al.* 2010), this fungus may have remained unremarked by the scientific community if it were not for its antibacterial producing properties.

In Sep. 1928, Fleming witnessed the contamination of one of his *Staphylococcus* plates by *Penicillium rubrum* and observed a halo of inhibition on his bacterial colonies (Fleming 1929). The antibiotic effect from this mould was *via* the production of the secondary metabolite penicillin, which was later refined for production by Ernst Chain and Howard Florey (Henk *et al.* 2011). These three scientists were acknowledged for their discovery by joint award of the Nobel Prize for Physiology or Medicine in 1945.

The taxonomy and true identity of Fleming's fungus was initially and has remained persistently controversial. The original penicillin-producing fungus was first described as *Penicillium rubum* by the experienced mycologist Charles La Touche (Fleming 1929), working downstairs from Fleming's laboratory (Henk *et al.* 2011; Houbraken *et al.* 2011). This identification was likely to be based upon comparison to fungal collections available to La Touche and a monograph of Biourge (1923). The epithet *rubrum* is derived from the Latin of ‘red’, and named accordingly due to the “reddish colour” as described by Fleming (1929). However, in 1930 another mycologist Charles Thom received Fleming's isolate for use in his latest monograph (Thom 1930) and reassigned the isolate from *P. rubrum* to *Penicillium notatum* (Thom 1945); a member of Thom's “*P. chrysogenum* series” (Thom 1910). Thom mistakenly criticised Fleming regarding the assignment to *P. rubrum* and commented, “not being a mycologist, he undertook to identify the mould from the literature and selected the name” (Thom 1945). Thom was apparently unaware that in fact La Touche had aided in the identification of Fleming's *Penicillium*. Nevertheless, the name *P. notatum* was carried forward until 1977 when, following the morphological study of isolate type strains, it was declared a synonym of *P. chrysogenum,* along with other species in Thom's series (Samson *et al.* 1977). Similarly, the older epithet *Penicillium griseoroseum* was synonymised with *P. chrysogenum* (Houbraken *et al.* 2011), however due to industrial importance of the name ‘*chrysogenum*’ it was suggested that ‘*griseoroseum*’ should be rejected (Frisvad *et al.* 1990). Since then, the name *P. chrysogenum* has been declared ‘*nomen conservandum*’, preserving the name and rejecting all predating epithets (Henk *et al.* 2011; Houbraken *et al.* 2011).

*P. chrysogenum* demonstrates that fungal species identification based on morphological and phenotypic characteristics can be challenging, often leading to contradictory findings and the grouping of more than one species (Taylor *et al.* 2000). As *P. chrysogenum* exhibits few distinguishing features, which additionally may vary depending on growth conditions and culture media (Henk *et al.* 2011). Using the genealogical concordance phylogenetic species recognition (GCPSR) for fungi has proven to be fruitful, by utilising multiple genealogies to detect and distinguish species by diagnosing breaks in concordance amongst gene phylogenies (Taylor *et al.* 2000). Phylogenetic studies have shown that there are multiple distinct lineages within what was previously considered *P. chrysogenum* (Scott *et al.* 2004; Henk *et al.* 2011). The analysis of 81 NRRL strains and 125 collected samples demonstrated the ‘Fleming species’, including the original Fleming isolate (NRRL-824) and the industrial penicillin producing strain (NRRL-1951, the isolate found on a mouldy cantaloupe), diverged from *P. chrysogenum sensu stricto* (NRRL-810) *circa* 0.8 million yr ago (MYA) (Henk *et al.* 2011). Furthermore, the authors described two additional species within the Chrysogenum species complex, provisionally named Species A and Species B, which are likely to have diverged even more recently from within the group (Henk *et al.* 2011). Species-specific diagnostic tools enabling discrimination between the four species are required as each species elicits minimal phenotypic features (Henk *et al.* 2011) which are likely to be unstable between different isolates. In this paper the term ‘Chrysogenum complex’ is applied rather than ‘Chrysogenum series’ as we are specifically referring to the four species recently described by Henk *et al.* (2011). The official ‘series Chrysogena’ additionally includes *Penicillium flavigenum, Penicillium dipodomyis* and *Penicillium nalgiovense* (Frisvad & Samson 2004).

In addition, Henk *et al.* (2011) discovered evidence of recombination in both *P. chrysogenum* and the ‘Fleming species’, and that species were composed of isolates of either MAT1-1 or MAT1-2 genotype, that were present in a near 1:1 ratio in sample populations. This suggested the presence of a ‘cryptic’ sexual cycle within these species (Henk *et al.* 2011). These data are supported by findings in the *P. chrysogenum* genome of mating-type genes, evidence of pheromone-signalling genes and the detection of a meiosis associated process, repeat induced point mutation (Hoff *et al.* 2008; van den Berg *et al.* 2008). We note that the isolate used for the ‘*P. chrysogenum*’ genome sequence was a strain derived from NRRL-1951 (Wisconsin-54-1255) and is now known to be of the ‘Fleming species’ (Henk *et al.* 2011; Houbraken *et al.* 2011). Recently, the genome of *P. chrysogenum senso stricto* has been sequenced (DOE Joint Genome Institute 2012).

The most recent phylogenetic analysis of a small collection of isolates has suggested that the ‘Fleming species’ should in fact be named as *Penicillium rubens* (Houbraken *et al.* 2011). This study placed the type strain of *P. rubens* (NRRL-792) within the ‘Fleming species’ clade (Houbraken *et al.* 2011), however, this type isolate was not included in the analysis by Henk *et al.* (2011) and should be included in a larger analysis. Houbraken *et al.* (2011) argued that, as this species already has a type isolate assigned the name *P. rubens*, this should take priority and be named so according to the rules of taxonomic nomenclature. The authors also suggested that the name *P. rubrum* be synonymised with *P. rubens* by Biourge and Thom (Thom 1930; Houbraken *et al.* 2011), and that actually La Touche may have been quite accurate in his identification of the original Fleming isolate.

In this study, we develop a diagnostic PCR tool for species identification and ecological study of the four known species in the Chrysogenum complex. After using molecular phylogenetic analysis to confirm the four species delimited by Henk *et al.* (2011), including *P. rubens* (NRRL-792), as the correct type for the ‘Fleming species’, we design four species-specific primer pairs for the diagnosis of the Chrysogenum complex species by PCR from purified DNA or directly from conidia. Based on our diagnostic, we name the previously undescribed Species A and B as identified by Henk *et al.* (2011), *Penicillium floreyi* and *Penicillium chainii* respectively. Finally, we apply our species-specific PCR tests in a preliminary exploration of the ecology of the Chrysogenum complex, through air sampling in London, St Mary's Hospital and the London Underground rail transport system (the Underground).

## Materials and methods

### Sampling of isolates

**Fig 1:**
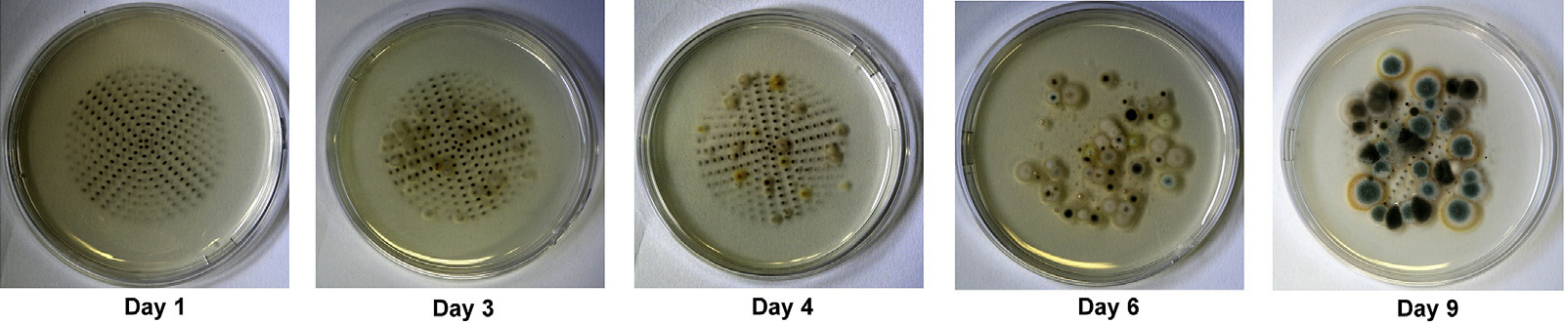
Air sample timeline. Photographs of different air sample plates over 9 d. *Penicillium* isolates, green colonies, are clearly visible at 9 d when PCR direct from conidia was performed along with fungal genera counts. Also visible at 9 d are the dark coloured colonies of the *Cladosporium* genus. Black particulate matter from underground station sample locations is clearly visible on the agar plates of 1, 3, 4 and 9 d. The 6 d plate was collected from an underground station with fewer particulates circulating in the air.

Sample isolates were collected using the MicroBio MB1 air sampler onto malt extract (MEA) or dichloran-glycerol with chloramphenicol (DG18) agar plates at 200, 500 or 1000 l of air. Nineteen locations across London were sampled including Fleming's laboratory and twelve stations on the Underground (GPS co-ordinates, Supplementary Text S1). Samples collected at Underground stations were taken on station platforms and all stations were located underground. Plates were maintained at room temperature until *Penicillium* isolates appeared (Fig 1) before being used for DNA extraction, PCR diagnostics or sequencing.

### DNA extraction, PCR and sequencing

Isolates used for DNA extraction were cultured at room temperature for 5 d on liquid broth MEA. All DNA extraction steps were performed using DNeasy Plant Mini Kits (Qiagen). The protocol (Qiagen 2006) was amended and optimised for fungal material by first homogenising 100 mg of fungal material using 0.5 mm glass beads in a Mini-BeadBeater (BioSpec), before continuation of the standard protocol.

Primers for multilocus sequence typing (MLST), *benA*, *trpC*, *crt1* and *ITS* used for PCR by Henk *et al.* (2011) were used in PCR with TopTaq PCR kits (Qiagen) and the following touchdown cycling protocol: 95 °C for 15 min, followed by 15 cycles of 95 °C for 30 s followed by a 65 °C annealing temperature for 30 s reduced by 1 °C in each repeated cycle followed by a 72 °C extension step for 45 s, followed by 35 cycles with a constant annealing temperature of 50 °C for 30 s and a final step of 72 °C for 4 min. An ABI 3 730 DNA sequencer was then used to sequence each PCR product using standard sequencing protocols (Godoy *et al.* 2003) and the same primers as used for PCR (Henk *et al.* 2011).

### Phylogenetic analysis

Eleven representative *Penicillium* spp. isolates and type isolates, including some not previously present in the analysis by Henk *et al.* (2011), were sequenced for each of the following four loci; *benA*, *trpC*, *crt1* and *ITS*. These isolates included *P. chrysogenum* isolate strains NRRL-792 (*P. rubens*), NRRL-834, NRRL-841, CBS-118871, CBS-101347, CBS-306.48 and CBS-302.67, and additionally, closely related ancestors of the Chrysogenum complex: *Penicillium confertum* (CBS-171.87), *Penicillium mononematosum* (CBS-172.87) and *P. flavigenum* (CBS-419.89 and CBS-110409). Alignments for the four loci were produced in MEGA 5 (Tamura *et al.* 2011) for a total of 217 isolate DNA sequences including the 11 sequenced isolates and a previous set of *P. chrysogenum* sequences from Henk *et al.* (2011) (*n* = 206, available from GenBank). Neighbour-joining phylogenetic trees based on maximum likelihood distances for each locus were produced and isolates were assigned to the four species as described by Henk *et al.* (2011). These species assignments were used for the species phylogeny technique developed by Heled & Drummond (2010) and applied using *BEAST and Beauti, parts of the BEAST package (Drummond *et al.* 2012). The *BEAST technique allows co-estimation of species phylogeny and population parameters using a mixture of coalescent and Yule processes (Heled & Drummond 2010). Thirty-seven isolates were selected for BEAST analysis, including the 11 newly sequenced isolates and 26 isolates from Henk *et al.* (2011). These isolates, sequenced for the four loci, were loaded into Beauti and used to generate an XML file of 1 000 000 generations for preliminary test runs. Using Tracer v1.5 to assess the runs, the XML was optimised in Beauti for a final run of 100 000 000 generations with parameters logged every 1000 generations. The HKY substitution model with empirical bases and invariant sites was implemented. To calculate divergence dates from the node heights, a molecular clock with fixed mean substitution rate of 3 × 10^−9^ per yr was used, as previously described for fungi of the class Eurotiomycetes (Kasuga *et al.* 2002). TreeAnotator was used to generate the maximum clade credibility tree after discarding the first 10 % of generations as burn-in and Tracer v1.5 was used to assess convergence.

### Primer design for species-specific PCR

DNA sequences (*n* = 206) of the *benA*, *parA*, *trpC* and *crt1* loci were retrieved from GenBank for the four species of *Penicillium* as identified by Henk *et al.* (2011). Alignments were performed using MEGA 5 (Tamura *et al.* 2011) to identify sites of variation between the four species allowing exclusive detection of one species per primer set. Primer Express Software v2.0 was then used to design forward and reverse primers targeting unique sites in each Chrysogenum complex species yielding amplicon lengths of 100–200 bases. The 3′ end of the primer was targeted to the unique species sites for maximum specificity.

DNA was extracted from four isolates representing the species of Henk *et al.* (2011) (NRRL-841, NRRL-792, NRRL-35692, NRRL-A1399), and two isolates representing out of group *Penicillium* species (CBS-171.89 and CBS-110409). We screened a variety of PCR conditions to optimise specificity for each primer pair using 25 μl reactions: 0.2 μl of TopTaq (Qiagen), 2.5 μl of ×10 TopTaq buffer, 0.4 μl 10 μM dNTP, 1 μl 10 μM of each primer, 17.9 μl HyPure Molecular Biology Grade Water and 2 μl of isolate 1:10 dilution DNA extract. Touchdown PCR cycling parameters were as follows: 95 °C for 15 min, followed by seven cycles of 95 °C for 30 s followed by a 72 °C annealing temperature for 30 s reduced by 1 °C in each repeated cycle (touchdown 72–65 °C), followed by a 72 °C extension step for 45 s, followed by 28–35 cycles (different between primer sets) with a constant annealing temperature of 65 °C for 30 s and a final step of 72 °C for 4 min. PCR products were subjected to electrophoresis in 2 % agarose gels.

*Penicillium* isolates (*n* = 148) collected through air sampling in London were tested by PCR using the four designed primer sets to ascertain primer specificity by observing for overlapping positives. To further confirm primer sensitivity and analyse *Penicillium* diversity, *ITS* regions from these 148 isolates were sequenced and assigned to species using the basic local alignment search tool (BLAST http://blast.ncbi.nlm.nih.gov/). Primer sets were also tested for efficacy when performing PCR direct from fungal conidia using an amended protocol (Aufauvre-Brown *et al.* 1993). Conidia from sample isolates were transferred direct into the PCR reactions by placing a pipette tip briefly in contact with an isolate colony from an agar plate and dipping into the reaction well. Conidia were also transferred directly into the PCR reactions from water backups of stored fungal isolates by the addition of 2 μl of backup water instead of the isolate DNA extract.

### Environmental detection and application of the species-specific primers

The application of the primer sets was demonstrated by air sampling and environmental detection of Chrysogenum complex fungi in London and the Underground. 430 *Penicillium* isolates collected from air sampling were tested by PCR using two of the designed primer sets detecting *P. chrysogenum* or the ‘Fleming species’. Species distributions between locations were statistically analysed in Microsoft Excel using the Chi-square test for independence, analysis of variance (ANOVA) and the Tukey–Kramer test, to significance *p* < 0.05.

Mating-type frequencies observed were tested against the null hypothesis of a 1:1 ratio using the Chi-square test for goodness-of-fit, to significance *p* < 0.05.

Fungal diversity was recorded from DG18 air sample plates at 9 d by counting total fungal colonies (Fig 1), *Penicillium* colonies, *Cladosporium* colonies, *Aspergillus* colonies, other genus colonies and yeast colonies. Genera counts were performed and repeated by a mycologist and an undergraduate.

### Determination of mating type

46 of the isolates collected were screened for mating type using MAT1-1 (MAT-1-f, MAT-1-r) and MAT1-2 (MAT-2-f, MAT-2-r) primer sets (Hoff *et al.* 2008). Following electrophoresis, isolates were assigned to either mating type if the expected amplicon band was detected exclusively in one of the primer sets (Hoff *et al.* 2008).

## Results

### Phylogenetics of the Chrysogenum complex

**Fig S1:**
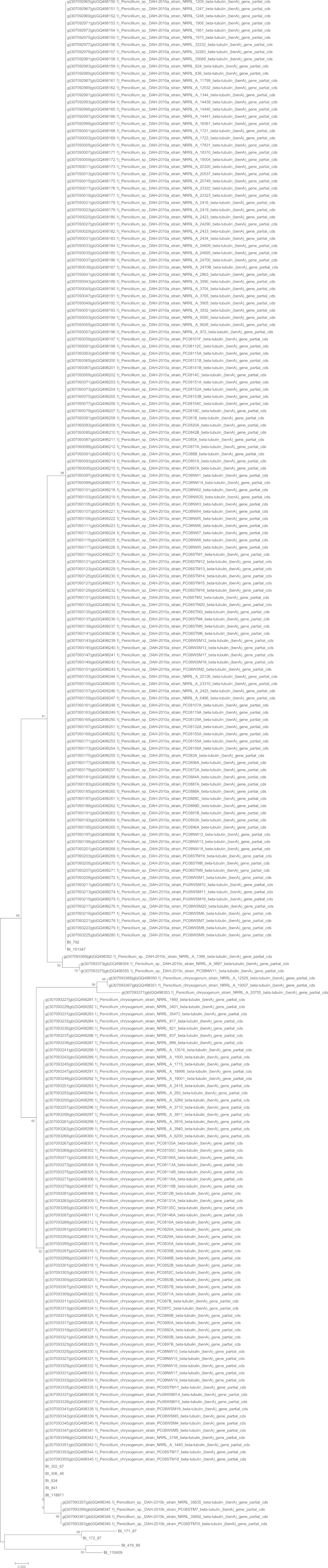
Neighbour-joining tree of the *benA* locus. This neighbour-joining phylogenetic tree produced in MEGA 5 (Tamura *et al.* 2011) shows all 217 isolates used to ascertain species assignments. Bootstrap values are shown.

**Fig S2:**
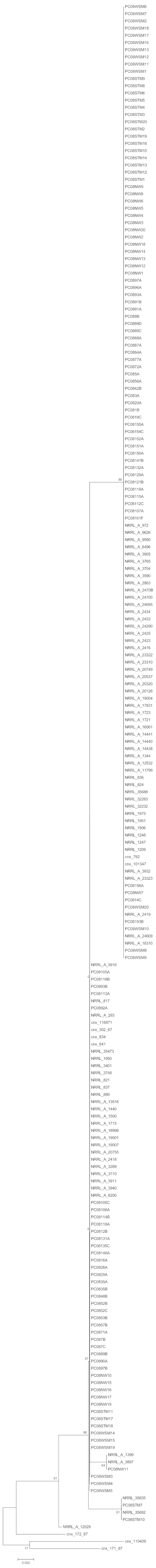
Neighbour-joining tree of the *crt1* locus. This neighbour-joining phylogenetic tree produced in MEGA 5 (Tamura *et al.* 2011) shows all 217 isolates used to ascertain species assignments. Bootstrap values are shown.

**Fig S3:**
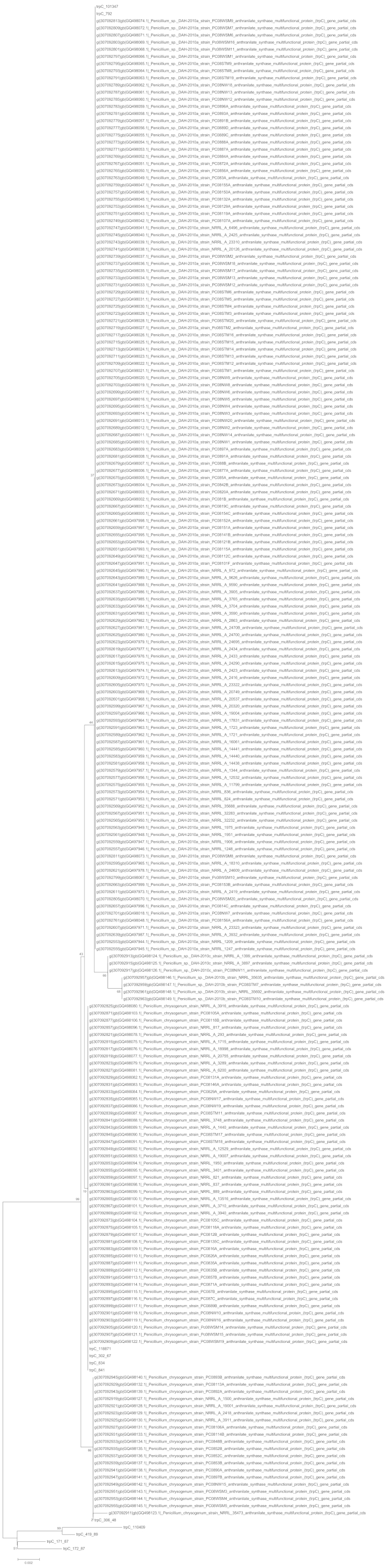
Neighbour-joining tree of the *trpC* locus. This neighbour-joining phylogenetic tree produced in MEGA 5 (Tamura *et al.* 2011) shows all 217 isolates used to ascertain species assignments. Bootstrap values are shown.

**Fig S4:**
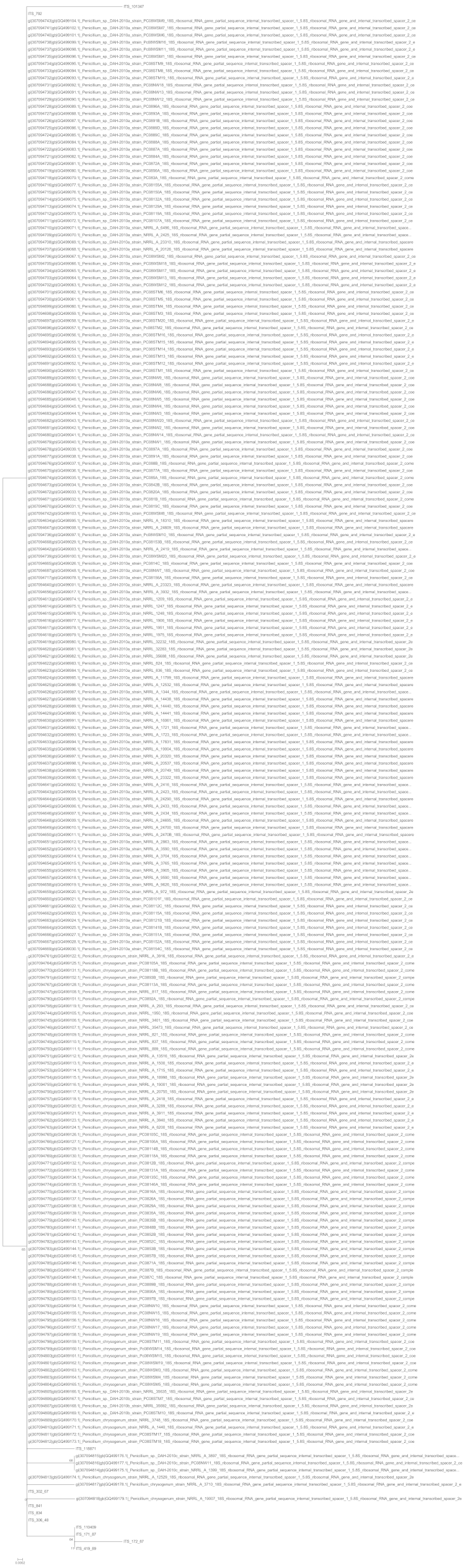
Neighbour-joining tree of the *ITS* locus. This neighbour-joining phylogenetic tree produced in MEGA 5 (Tamura *et al.* 2011) shows all 217 isolates used to ascertain species assignments. Bootstrap values are shown.

**Fig 2:**
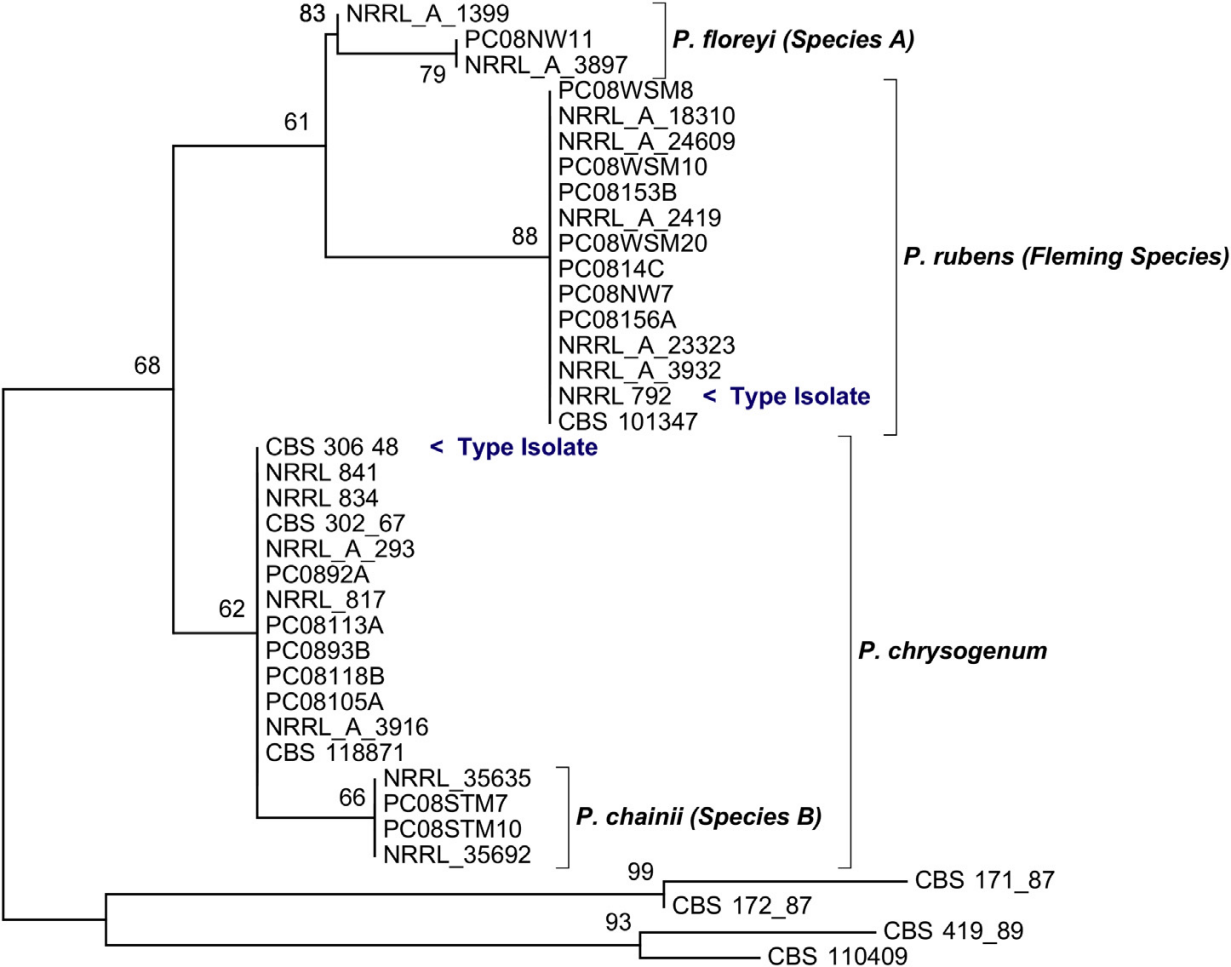
Neighbour-joining tree of the *benA* locus. This neighbour-joining phylogenetic tree produced in MEGA 5 (Tamura *et al*. 2011) shows the selected isolates (37/217) used for *BEAST analysis. All four loci (*benA*, *crt1*, *trpC* and *ITS*) trees demonstrated the same results (Figs S1–4). Here we can see the type isolates of *P. rubens* and *P. chrysogenum* falling into their respective species. Type isolates were not assigned for species A and B. Closely related ancestors of the Chrysogenum complex were excluded from the four species groups; *P. confertum* (CBS-171.87), *P. mononematosum* (CBS-172.87) and *P. flavigenum* (CBS-419.89 and CBS-110409). Bootstrap values are shown.

The same species assignments were seen in all four loci neighbour-joining trees (Fig 2, Figs S1–4). NRRL-792 and CBS-101347 were assigned to the ‘Fleming species’ as described by Henk *et al.* (2011), and NRRL-834, NRRL-841, CBS-302.67, CBS-306.48 and CBS-118871 were assigned to the *P. chrysogenum* species. Close ancestors of the Chrysogenum complex were excluded from the four species groups as expected.

**Fig S5:**
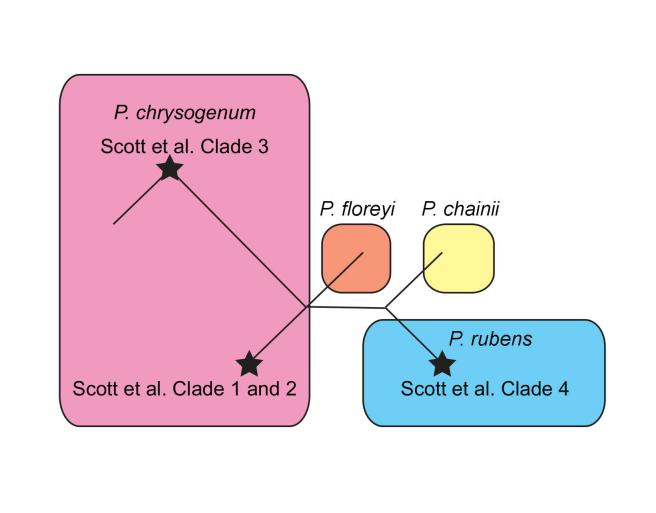
Species comparison to the Scott *et al.* clades. Scott *et al.* 2004 described four clades in *P. chrysogenum senso lato*. In this figure we display how these clades previously described fit into the four species in the Chrysogenum complex.

Consistent with the findings of Houbraken *et al.* (2011), the type strain of *P. rubens*, NRRL-792, was placed within the ‘Fleming species’, and this species is correctly named *P. rubens*. Species A and Species B described by Henk *et al.* (2011) were not accounted for by a type strain and, therefore, will be diagnosed and formally assigned new taxonomic names in this paper. Species A will be named *P. floreyi* and Species B named *P. chainii* (see section on Taxonomy). The species here have been compared to the clades proposed by Scott *et al.* 2004 (Fig S5). *P. chrysogenum* includes Scott clades 1, 2 and 3, *P. rubens* is Scott clade 4 and the two new species were not described under the Scott clades.

**Fig S6:**
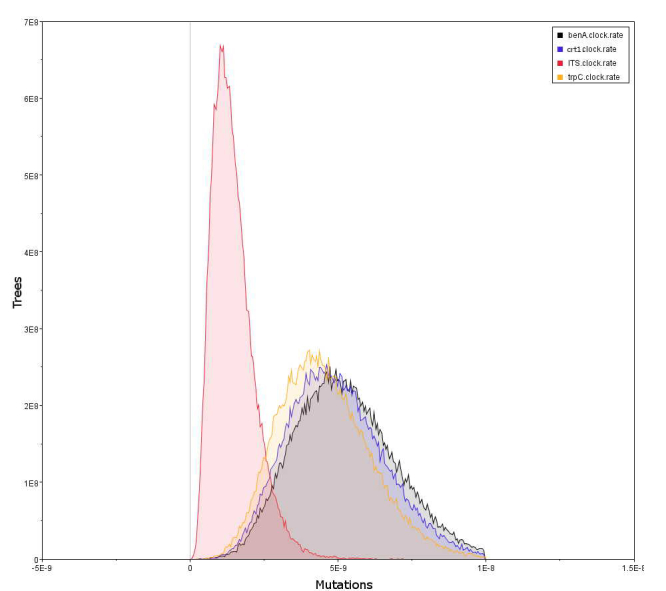
Mutation rates of MLST loci. Generated using Tracer v1.5, this histogram shows the distribution of different mutation rates present in the four loci used for *BEAST analysis. The non-functional *ITS* locus demonstrated slow mutation as it is highly conserved as a spacer for rRNA. This *ITS* mutation rate corresponds to the findings of Kasuga *et al.* 2002.

*BEAST analysis resulted in large effective sample sizes (ESS = 476–48 517) with convergence on all parameters. The mutation rate for *ITS* equalled 1.46 × 10^−9^ yr^−1^ compared to 4.54–5.23 × 10^−9^ for the other three loci (Fig S6). The species Yule birth rate was 6.7 × 10^−7^ per yr (0.67 new species every 1 million yr).

**Fig 3:**
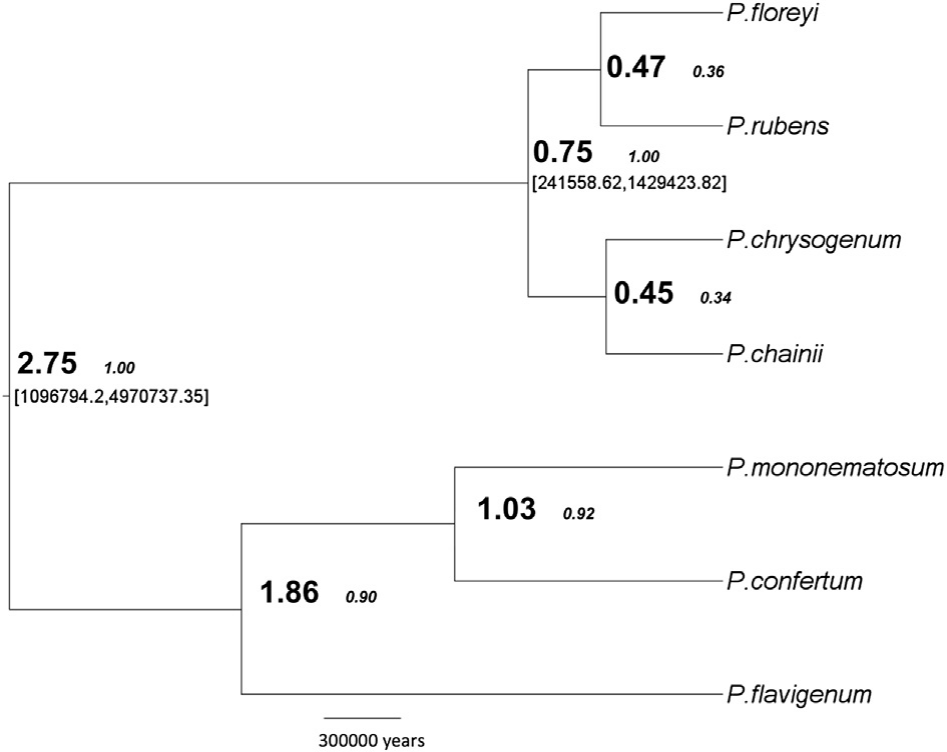
Phylogenetics and divergence of the Chrysogenum complex. Maximum-clade-credibility phylogenetic tree of the combined *benA*, *trpC*, *crt1* and *ITS* loci from *BEAST analysis. [95 % HPD] values are shown in brackets. Mean node ages (**MYA**) are shown in bold with posterior values (*1.00*) in small italics.

Unlike Henk *et al.* (2011), we included closely related ancestors of the Chrysogenum complex. Divergence of Chrysogenum complex species from the ancestors, *P. flavigenum*, *P. mononematosum* and *P. confertum* occurred 2.75 MYA (95 % highest posterior density (HPD) = 1.1 × 10^6^–5.0 × 10^6^), supported by posterior probability value of 1 (Fig 3). *P. flavigenum* was ancestral to both *P. mononematosum* and *P. confertum* with divergence occurring 1.86 MYA. The Chrysogenum species complex diverged 0.75 MYA (HPD = 2.4 × 10^5^–1.4 × 10^6^) supported by posterior value of 1 (Fig 3). We demonstrate a divergence time comparable to that of Henk *et al.* (0.8 MYA) using a different data set including close ancestors of the Chrysogenum complex and additional type isolates, such as *P. rubens* (NRRL-792). Our suggested divergence of the four Chrysogenum complex species was into two main branches with *P. rubens* and *P. floreyi* diverging in branch one, and *P. chrysogenum* and *P. chainii* diverging in branch two. Posterior values for these divergences were 0.36 and 0.34 respectively; therefore, the relative positioning of divergences cannot be stated with certainty, although, the mean node ages suggest divergence 0.47 and 0.45 MYA.

### Species-specific primers for *P. chrysogenum*, *P. rubens*, *P. chainii* and *P. floreyi*

**Fig 4:**
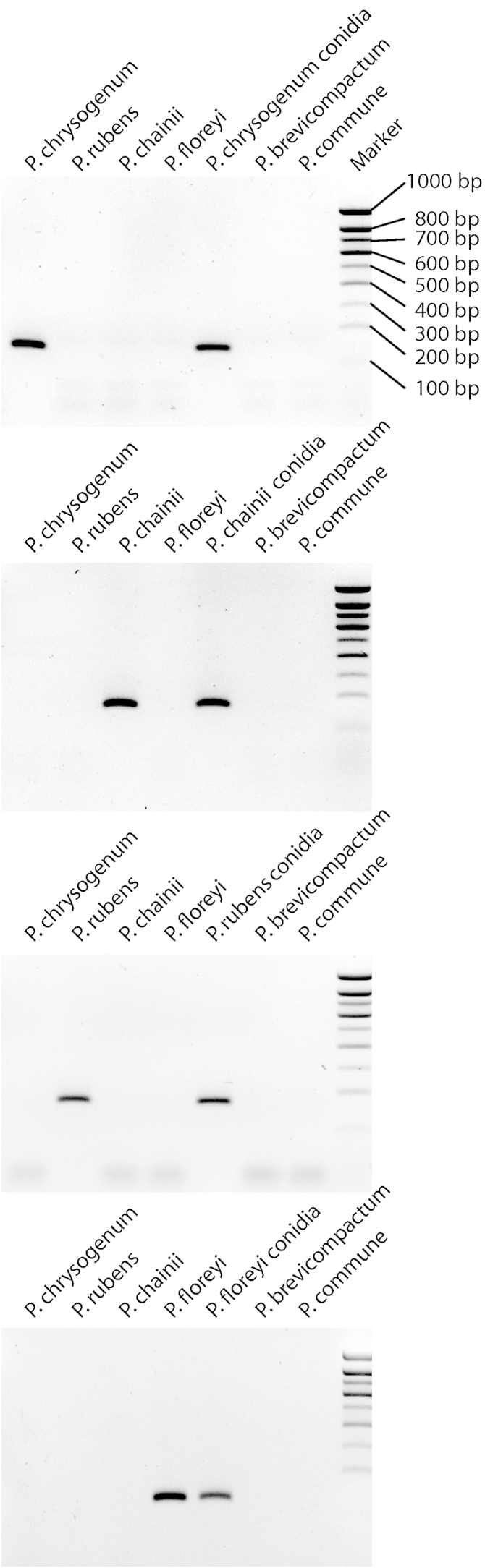
Species-specific PCR primers for the Chrysogenum complex. Electrophoresis gel (2 % agarose) under UV light demonstrating species-specificity for each primer set (four panels) from DNA extracts. Expected amplicon bands are visible between 100 and 200 base pairs (bp) depending on the primer set (Table 1). Amplicons from PCR direct from conidia were detected and show additional specificity when tested against other common airborne conidia of the species *P. commune* and *P. brevicompactum*.

Multiple primer sets were synthesised and optimal specificity was exhibited in the following primers: benA_Chrysogenum, crt1_Rubens, crt1_Chainii and parA_Floreyi (primer set sites, Text S2). These species-specific diagnostics tools (Table 1) are able to discriminate between the four species identified by Henk *et al.* (2011) (Fig 4), allowing their relative distributions in the environment to be described.

PCR direct from fungal conidia was successful in all four primer sets using the same PCR conditions as from pure DNA extracts and yielding the same efficacy including discrimination of other common airborne *Penicillium* species (Fig 4). Sequencing of the *ITS* region was also successfully performed direct from conidia. 148 isolates tested by all primer sets showed no overlapping positives and BLAST results from these isolates' *ITS* sequences did not reveal any undetected isolates within the Chrysogenum complex. All isolates indicated as Chrysogenum complex by BLAST results were accounted for by being PCR positive for either *P. chrysogenum* or *P. rubens*.

Based on rapid discrimination the 430 *Penicillium* isolates recovered by air sampling and tested by PCR from conidia, 58 (13.5 %) isolates were found to be *P. chrysogenum* and 65 (15.1 %) isolates *P. rubens*. There was no overlapping of positive results, adding further evidence that these primers are species-specific. Of the 430 isolates, a sub-sample of 148 were tested for *P. chainii* and *P. floreyi,* however, no positives were detected confirming the rarity of these two species. The locations sampled were grouped into five similar environments; the varied populations of *P. chrysogenum* and *P. rubens* are displayed in Table 2.

No significant difference (*p* > 0.05) was found between the numbers of *P. chrysogenum* and *P. rubens* detected in MEA and DG18 samples taken at the same location: Bakerloo Line (*p* = 0.529), St Mary's Hospital (*p* = 0.389) and the Outdoors (*p* = 0.308), nor between 500 and 1 000 l of air sample volume (*p* = 0.071). This allowed the data set to be statistically analysed together to compare sample environments.

ANOVA revealed no significant difference in the distribution of *P. chrysogenum* between locations (*p* = 0.173) but did for *P. rubens* (*p* = 0.002). Post-hoc analysis by the Tukey–Kramer test revealed significant difference was located. There was a statistical difference (*p* < 0.05) in the number of *P. rubens* between the Central and Bakerloo Lines, and between the Central Line and the Outdoors.

The Chi-square test revealed no significant difference (*p* > 0.05) in the ratio of *P. chrysogenum* to *P. rubens* between locations (Table 3). However, significant differences were found between multiple locations in the ratios of *P. chrysogenum* to other *Penicillium*, *P. rubens* to other *Penicillium*, and total Chrysogenum complex *Penicillium* to other *Penicillium*. Both the Central and Jubilee Lines were statistically different to the Outdoors for all three ratio comparisons. The Bakerloo and Central Line were also significantly different for two ratio comparisons and borderline significant for the third.

Sequencing of the *ITS* for 148 *Penicillium* isolates revealed that 57 (38.5 %) were similar to *Penicillium brevicompactum*, suggesting it to be the most common *Penicillium* in London. Sequencing also revealed that 33 (22.3 %) isolates were similar to *P. chrysogenum*, and that 32 (21.6 %) were similar to *Penicillium commune*.

**Fig 5:**
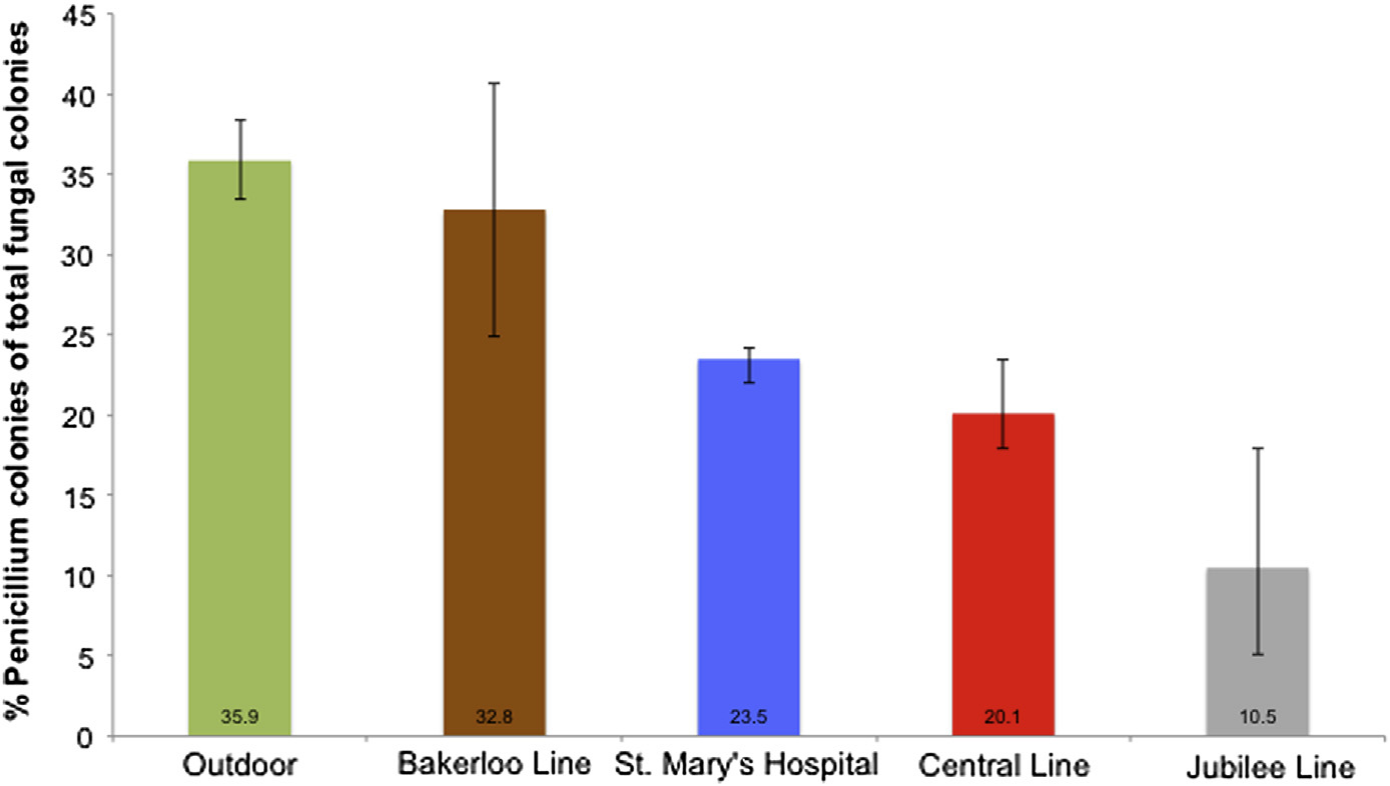
Varying proportions of *Penicillium* across the underground and other London locations. Percentages shown are means of all samples taken from that location. Error bars display the range of proportions measured at the sample sites within each location.

Fungal genera counts displayed varying proportions of *Penicillium* colonies between locations (Fig 5). On the Underground, the Jubilee Line had the lowest number of *Penicillium* colonies as a percentage of total fungal colonies with 10.5 %, followed by the Central Line with 20.1 % and the Bakerloo Line with 32.8 %.

The number of viable fungal conidia inhaled per minute also varied across locations (Table 4). St Mary's Hospital had the fewest conidia with 0.26 conidia per minute whereas the Underground had an average of 0.98.

Mating-type diagnostics detected MAT1-1 and MAT1-2 isolates in both *P. chrysogenum* (13:11) and *P. rubens* (8:14). 1:1 ratios could not be rejected for either species, *p* = 0.68 and *p* = 0.20 respectively.

#### Taxonomy

***P. floreyi***
Henk, A. G. P. Browne et Fisher sp. nov.
[MycoBank: MB515082]

Description: Colonies on MEA velvety with some floccose growth; forming dull, grey, or dark green regions of conidiation; occasional production of exudates. Produces a halo of inhibition of *Staphylococcus aureus* growth between 2 and 3 cm from the leading edge of the fungal colony after 5 d of growth. Conidiophores mostly terverticilliate, divaricate. Stipes smooth and 100–350 μm × 3–5 μm, Phialides ampulliform and closely packed. Conidia smooth, globose to subglobose and 2–4 μm in diameter.

Diagnosis: *Penicillium floreyi* is similar to *P. chrysogenum*, *P. chainii*, and *P. rubens* based on colony and conidial characters when grown on standard media. It is distinct from the other species in the Chrysogenum complex following the polymerase chain reaction using primers parA_floreyi targeting the *parA* region (5′—ACGGCCCCTCCTTACGAAA—3′, 5′—TGTGAGACCAAAGGCAGTGG —3′). Detection of *P. floreyi* is demonstrated following gel electrophoresis of the PCR product by a visible band at approximately 120 bp.

Distribution: United States of America, United Kingdom.

HOLOTYPE: K(M)163135, a dried culture derived from NRRL-A1399 isolated from cloth in Florida, USA. Ex-holotype culture: NRRL-A1399. Other strains examined: PC08NW11 (UK) and NRRL-3897 isolated from Ohio, USA.

Etymology: floreyi; after Howard Florey who along with Fleming and Chain won a Nobel prize for his work isolating penicillin from this complex of fungi.

***P.chainii***
Henk, A. G. P. Browne et Fisher sp. nov.
[MycoBank: MB515083]

Description: Colonies on MEA velvety; forming grey-, or grey-green regions of conidiation; occasional production of exudates. Produces a halo of inhibition of *S. aureus* growth between 2 and 4 cm from the leading edge of the fungal colony after 5 d of growth. Conidiophores mostly terverticilliate, divaricate. Stipes smooth and 100–300 μm × 3–5 μm, Phialides ampulliform and closely packed. Conidia smooth, globose to subglobose and 2–4 μm in diameter.

Diagnosis: *Penicillium chainii* is similar to *P. chrysogenum*, *P. floreyi*, and *P. rubens* based on colony and conidial characters when grown on standard media. It is distinct from the other species in the Chrysogenum complex following the polymerase chain reaction using primers crt1_chainii targeting the *crt1* region (5′—CTTTCTACAATTGCTCGCGTTTTTATTTG—3′, 5′—CCTTGTTAGTGGCACCGCACTTA—3′). Detection of *P. chainii* is demonstrated following gel electrophoresis of the PCR product by a visible band at approximately 180 bp.

Distribution: France, Portugal, United Kingdom.

HOLOTYPE: K(M)163134, a dried culture derived from NRRL-35635 isolated from cork in Portugal. Ex-holotype culture: NRRL-35635. Other strains examined: PC08STM7 (UK), PC08STM10 (UK) and NRRL-35692 isolated from grape in France.

Etymology: chainii; after Ernst Chain who along with Fleming and Florey won a Nobel prize for his work isolating penicillin from this complex of fungi.

## Discussion

Our phylogenetic analysis adds further evidence to support the name *P. rubens* for the ‘Fleming species’ owing to the existence of a type isolate (NRRL-792) with nomenclatural priority (although the type assigned was a neotype designated by later authors who considered it a synonym of *P. chrysogenum*). However, Species A and B were not accounted for by known type isolates, demonstrating the novelty of these species. Here, based on our molecular diagnostic assay, they have been assigned type isolates (NRRL-A1399 and NRRL-35635) and are formally named *P. floreyi* and *P. chainii* respectively. We conclude that the divergence of the Chrysogenum complex occurred ≈0.75 MYA with more recent divergences arising ≈0.5 MYA; however the precise relative positioning of species could not be inferred with strong statistical support. Identifying the relative branching order amongst these four cryptic species may require the analysis of much larger datasets, whole genome sequences, or may not be possible. We designed species-specific diagnostics for *P. chrysogenum*, *P. rubens*, *P. chainii* and *P. floreyi* allowing rapid detection via PCR direct from conidia. We then applied our diagnostic tools by air sampling in London and the Underground to demonstrate fast environmental detection of Chrysogenum complex fungi. This tool confirmed that both *P. chrysogenum* and *P. rubens* are common fungi with their distributions varying between sample locations. Additionally, MAT1-1 and MAT1-2 were detected in both species at a ratio of near 1:1 adding further evidence for on-going recombination in the Chrysogenum complex. Finally, we detected a greater proportion of fungal conidia on the Underground compared to outdoor sample sites, and found that the proportions of *Penicillium* species present varied between Underground lines.

### The true identity of Fleming's famous fungus

The *nomen conservandum* was placed upon the name *P. chrysogenum* (neotype strain, CBS-306.48 = NRRL-807) to maintain the widely used epithet in numerous applications, including the patents for the $8 billion industry (Hoff *et al.* 2008) of penicillin production (Frisvad *et al.* 1990; Kozakiewicz *et al.* 1992). However, by including the isolate NRRL-792 in phylogenetic analysis, we and others (Houbraken *et al.* 2011) have shown that the species containing Fleming's original isolate (NRRL-824) and the isolate from which all industrial strains are derived (NRRL-1951) is in fact *P. rubens*. The implication of this is that it may seemingly invalidate some existing patents referring to *P. chrysogenum*, and thus, should these patents be amended accordingly? Another option available could be to designate a neotype for *P. chrysogenum* using one of the ‘Fleming species’ isolates. This would in effect rename *P. rubens* to *P. chrysogenum* in order to carry out the original objective of the *nomen conservandum*. It seems that this approach would be most convenient for industrial matters but rather confusing and backward with respect to taxonomic science. Additionally, and as previously stated by Henk *et al.* (2011), “designation of a neotype when a clear, intact and available type is known, could prevent this approach”. This leads us to question the quality of the *P. rubens* type strain NRRL-792. Unfortunately, NRRL-792 is a lectotype identified from phenotypic characteristics from Biourge's original illustrations (Biourge 1923; Thom 1930; Raper & Thom 1949; Houbraken *et al.* 2011). As we have seen from the problems associated with morphological species identification (Taylor *et al.* 2000), it cannot be assured that NRRL-792 is actually the species first described by Biourge. His other closely related species have all been synonymized with *P. chrysogenum* both here and previously. This illustrates the uncertainty surrounding the assignment of type specimens based purely on phenotypic characteristics and is a reoccurring problem in fungal taxonomy. The options available to taxonomists to resolve these issues may invalidate industrial patents and people may argue that the continuous separation of species is confusing matters more than it is driving science. The taxonomy discussed will be considered further following dissemination of this research.

### Fungi of the London Underground

By using our species-specific PCR tool, we have started to explore the relative distributions of these species within the Underground rail system. We focussed on the populations of *P. chrysogenum* and *P. rubens* (430 isolates tested) due to the ubiquity of these species and left the ecology of *P. chainii* and *P. floreyi* largely unexplored (148 isolates tested).

Much of the research towards *P. chrysogenum* and *P. rubens* has concentrated on penicillin production and the genetics of secondary metabolism, leaving their ecology and population biology mostly unexplored. The main studies to date have shown *P. chrysogenum* to be the most frequent and ubiquitous *Penicillium* when analysing indoor samples (Beguin & Nolard 1994; Scott 2001), whilst seeming to be rarer when analysing outdoor samples (Scott *et al.* 2004). Since the description of the four Chrysogenum complex described by Henk *et al.* (2011), there has been no further research into their relative distributions and populations. The London Underground carries a total of 1107 million passengers per yr over a network of 249 miles (Transport for London 2012). Within the network, 45 % of the train lines are contained within tunnels, with the deepest, the Jubilee Line, reaching a mean depth of 32 m below sea level (Transport for London 2012). The Underground is a unique and heterogeneous environment that differs greatly from environments on the surface. Due to these features, evidence suggests that the Underground has even hosted speciation of the *Culex pipiens* mosquito (Byrne & Nichols 1999). Fungal diversity has also been studied on the Underground with significant differences found between stations (Gilleberg *et al.* 1998) and similar fungal studies on underground stations in Milan, Cairo and St Petersburg have also demonstrated interesting results (Picco & Rodolfi 2000; Awad 2002; Bogomolova & Kirtsideli 2009). Further study into the diversity and population biology of fungi of the Underground environment may have health implications towards the evident link between fungal allergens and asthma (Beguin & Nolard 1994; Gilleberg *et al.* 1998; Denning *et al.* 2006). *P. chrysogenum* is an important human allergen in its own right, with environmental exposure shown to induce and exacerbate atopic asthma in sensitive people (Shen *et al.* 2003; Denning *et al.* 2006). Additionally, *P. chrysogenum* is the most commonly used species in the clinical diagnosis of *Penicillium* allergy (Shen *et al.* 2003).

Although there are proven links between indoor mould exposure and asthma (Denning *et al.* 2006), the increased fungal exposure on the Underground is highly unlikely to affect healthy individuals. Those already suffering with asthma may be more likely to suffer respiratory symptoms in any ‘mouldy location’ and may already be sensitive to *P. chrysogenum* (Denning *et al.* 2006). Similarly, particulate matter present in the air of the Underground was clearly visible on our sample plates (Fig 1), however, these Underground particulates, mainly consisting of iron oxide, are thought to be relatively safe for people at low respiratory risk (Colvile 2005; Seaton *et al.* 2005). Additionally, the recent diversity found within the Chrysogenum complex may have implications regarding known fungal allergens produced by *P. chrysogenum* (Denning *et al.* 2006).

The differences in fungal populations observed between locations, such as Underground lines, could be the result of local-scale environmental variation. A diagrammatic map by Transport for London (2010) highlights variability of air temperature between Underground lines; particularly in the coolness of the Jubilee Line. The lower temperatures, around 24 °C, recorded here could partly explain why lower proportions of *Penicillium* were seen on the Jubilee Line, England to the Bakerloo and Central. Fitting with this hypothesis, the temperatures on the Bakerloo and Central range from around 31–35 °C and genera counts revealed increased *Penicillium* proportions. However, the data displayed on the diagrammatic map were collected during a short 4 d window in Jul. 2010 between the hr of 1600 and 1800. Although this temperature survey is very limited, similar patterns in temperatures are likely to be observed outside these time periods and remain relative between different lines. The Milan underground study specifically provides data showing *Penicillium* colonies directly correlating with rise in temperature (Picco & Rodolfi 2000). The Cairo metro study reports slightly higher proportions of *Penicillium* at an underground station than that of an overground station (Awad 2002). *Penicillium* populations in the Underground are known to vary between stations (Gilleberg *et al.* 1998) and our data support this. It has been suggest that the fungi present are more likely to be “indicative of local moulding” rather than transportation of conidia from outdoor air (Gilleberg *et al.* 1998).

Our results and previous research demonstrate evidence that the distributions and populations of fungi are different on the Underground to that above ground. The diagnostic tool we developed and applied has provided some interesting preliminary data and will inform future investigations into the ecology of fungal communities in Underground rail networks. As the primary aim of this research was to prove the efficacy and application of our diagnostic tools, the statistical analysis of sample environments in this study has some limitations. First, the concentration of airborne fungi can vary throughout the day along with change of weather. Second, due to the nature of air sampling, more than one spore can be impinged at a single point onto the culture media leading to a false reflection of the true number of fungal colonies. Additionally, we used two different culture media and three air sample volumes; however, we found no statistical difference between these variables. Taking these limitations into account, we have nonetheless demonstrated significant differences between sample environments which need further investigation. Research primarily designed to explore the poorly understood ecology of Chrysogenum complex could utilise our species-specific PCR diagnostic for location comparison.

Mating types in a near 1:1 ratio present in both *P. chrysogenum* and *P. rubens* provide further evidence to support sexual reproduction. The sample sizes (24 and 22 respectively) used for the mating-type distribution analyses are relatively small compared to the larger analyses within this paper, therefore, firm conclusions cannot be drawn. However, the sexual reproduction of *Penicillium* has recently received increasing interest due to the scope of having the ability to artificially mate strains in the laboratory (Hoff *et al.* 2008; Dyer & O'Gorman 2011). Although this has been similarly achieved in *Aspergillus* (O'Gorman *et al.* 2009), it has not yet been observed in *Penicillium* which, if achieved, may have implications for production of penicillin or other metabolites by means of sexually improving strains (Hoff *et al.* 2008; Dyer & O'Gorman 2011).

Finally, it seems suitable to conclude by mentioning that air samples from Fleming's Laboratory contained more *P. rubens* than any other location sampled in St Mary's Hospital. Although our findings may show Alexander Fleming to not have been particularly lucky in observing *P. rubens*, further exploration of the natural ecology of *P. rubens* and its closest relatives may yet reveal underlying causes of Fleming's observation. The ancillary roles of the scientists Chain and Florey, who translated Fleming's observations and research into medical practice, are recognised in the names of our new species.

## Figures and Tables

**Table 1:** Species-specific primer sets with optimum PCR cycles are shown in this table. All primer sets demonstrate specificity at 65 °C annealing temperature, a full description of the touchdown PCR cycle is available in the Materials and Methods section. Gel electrophoresis of the PCR products reveals visible bands at the expected amplicon lengths (Fig 4)

Species-specificity	Primer set name	Amplicon length	Forward primer 5′–3′	Reverse primer 5′–3′	Number of cycles
*P. chrysogenum*	benA_Chrysogenum	111	CATGTGAGTACAATGACTGGGAATCTT	TCGACCAGAACGGCACG	35
*P. rubens*	crt1_Rubens	180	CCTCGAGGACTTCAATCCTCCCGTG	GTCAGCGGGCTGAGTGGCC	28
*P. chainii*	crt1_Chainii	185	CTTTCTACAATTGCTCGCGTTTTTATTTG	CCTTGTTAGTGGCACCGCACTTA	35
*P. floreyi*	parA_Floreyii	111	ACGGCCCCTCCTTACGAAA	TGTGAGACCAAAGGCAGTGG	32

**Table 2:** Distribution of 430 isolates of *P. chrysogenum* and *P. rubens* in London and on the underground

Location group	Location	Total number of *Penicillium* isolates	Number of *P. chrysogenum*	(%)	Number of *P. rubens*	(%)	Number of other *Penicillium*	(%)
Bakerloo Line	Baker street	22	5	(22.7)	0	(0.0)	17	(77.3)
Edgware road	31	4	(12.9)	3	(9.7)	24	(77.4)
Marylebone	40	3	(7.5)	5	(12.5)	32	(80.0)
Paddington	39	5	(12.8)	3	(7.7)	31	(79.5)
Bakerloo Line total	132	17	(12.9)	11	(8.3)	104	(78.8)
Central Line	Chancery lane	30	5	(16.7)	6	(20.0)	19	(63.3)
Holborn	27	7	(25.9)	9	(33.3)	11	(40.7)
Oxford circus	35	5	(14.3)	6	(17.1)	24	(68.6)
Tottenham court road	33	6	(18.2)	9	(27.3)	18	(54.5)
Central line total	125	23	(18.4)	30	(24.0)	72	(57.6)
Jubilee Line	Bond street	32	9	(28.1)	6	(18.8)	17	(53.1)
Green park	17	0	(0.0)	2	(11.8)	15	(88.2)
Waterloo	8	0	(0.0)	2	(25.0)	6	(75.0)
Westminster	16	1	(6.3)	4	(25.0)	11	(68.8)
Jubilee line total	73	10	(13.7)	14	(19.2)	49	(67.1)
St Mary's Hospital	Hospital corridor	12	3	(25.0)	0	(0.0)	9	(75.0)
A&E	16	0	(0.0)	2	(12.5)	14	(87.5)
Alexander Fleming's lab	20	3	(15.0)	4	(20.0)	13	(65.0)
DIDE laboratory	4	0	(0.0)	1	(25.0)	3	(75.0)
St Mary's hospital total	52	6	(11.5)	7	(13.5)	39	(75.0)
Outdoors	Norfolk square gardens	16	1	(6.3)	2	(12.5)	13	(81.3)
Canal	16	1	(6.3)	1	(6.3)	14	(87.5)
Praed street	16	0	(0.0)	0	(0.0)	16	(100.0)
Outdoors total	48	2	(4.2)	3	(6.3)	43	(89.6)
Grand total		430	58	(13.5)	65	(15.1)	307	(71.4)

Statistical difference (using the Tukey–Kramer test following ANOVA, *p* < 0.05) in the number of *P. rubens* was found between the Central and Bakerloo Lines, and between the Central Line and the Outdoors.

**Table 3:** Chi-square analysis of differences in *Penicillium* distribution ratios between the underground and London locations

Ratio comparison^a^	Location	Central Line	Jubilee Line	Outdoors	St Mary's hospital
C:R	Bakerloo Line	0.138	0.171	0.388	0.382
Central Line	X	0.887	0.883	0.858
Jubilee Line	X	X	0.945	0.793
Outdoors	X	X	X	0.814
C:O	Bakerloo Line	0.056	0.609	0.084	0.905
Central Line	X	0.286	*0.004	0.138
Jubilee Line	X	X	*0.048	0.613
Outdoors	X	X	X	0.138
R:O	Bakerloo Line	*0.000	*0.020	0.536	0.304
Central Line	X	0.310	*0.002	0.065
Jubilee Line	X	X	*0.026	0.360
Outdoors	X	X	X	0.180
C + R:O	Bakerloo Line	*0.000	0.066	0.098	0.578
Central Line	X	0.185	*0.000	*0.029
Jubilee Line	X	X	*0.005	0.342
Outdoors	X	X	X	0.058

*p* values (<0.05 highlighted by *) from the Chi-square test for independence indicate differences in the ratios of *Penicillium* between locations. No statistical difference was shown between locations in the ratio of *P. chrysogenum* to *P. rubens*. The Central and Jubilee Line were consistently different to the Outdoors in the other *Penicillium* ratios.

a Ratio comparison abbreviations, C = *Chrysogenum*, R = *Rubens*, O = Other *Penicillium*.

**Table 4:** Estimates of inhaled fungal conidia at locations in London. Using the total fungal colonies counted and the air volume sampled, the number of viable conidia inhaled per minute was calculated at each location for humans. A tidal volume of 0.5 l and respiratory rate of 15 breaths per minute was used

Location	Viable conidia inhaled per min
Jubilee Line	1.11
Central Line	1.09
Bakerloo Line	0.73
Outdoors	0.53
St Mary's hospital	0.26

## Footnotes

Supplementary data related to this article can be found at http://dx.doi.org/10.1016/j.funeco.2013.04.003.
