# Supplementary material for: Species-specific PCR to describe local-scale distributions of four cryptic species in the Penicillium chrysogenum complex
Source: Fungal Ecol. 2013 Oct;6(5):419–29. doi: 10.1016/j.funeco.2013.04.003 (PMC3809933; doi:10.1016/j.funeco.2013.04.003)
Supplement: Supplementary file 1 [file mmc1.docx]

**Supporting Information**

**Text S1 – Sample location GPS co-ordinates**

1. Outdoors
   1. **Praed Street** 51.516998,-0.173019
   2. **Norfolk Square Gardens** 51.515587,-0.174097
   3. **Canal** 51.518295,-0.173475
2. St Mary’s Hospital
   1. **Alexander Fleming’s Lab** 51.517082,-0.173094
   2. **DIDE Laboratory** 51.517723,-0.173137
   3. **Hospital Corridor** 51.517326,-0.173590
   4. **A&E** 51.517857,-0.174847
3. Bakerloo Line Stations
   1. **Paddington** 51.515842,-0.176229
   2. **Edgware Road** 51.520283,-0.170159
   3. **Marylebone** 51.522213,-0.163170
   4. **Baker Street** 51.523045,-0.156997
4. Central Line Stations
   1. **Tottenham Court Road** 51.516235,-0.130883
   2. **Holborn** 51.517368,-0.120262
   3. **Chancery Lane** 51.518120,-0.111528
   4. **Oxford Circus** 51.515141,-0.141912
5. Jubilee Line Stations
   1. **Bond Street** 51.514366,-0.149594
   2. **Green Park** 51.506874,-0.142985
   3. **Westminster** 51.501102,-0.124939
   4. **Waterloo** 51.503185,-0.115047

**Text S2 – Primer set sites**

***Penicillium chrysogenum***

benA_Chrysogenum

Amplicon length - 111 bases

Forward primer - site 216/217

CATGTGAGTACAATGACTGGGAATCTT

Chrysogenum TCAACCATGTGAGTACAATGACTGGGAATC**_ _**TTAATTGTGCATCATCTGATCGGGCGTTT

Rubens TCAACCATGTGAGTACAATGACTGGGAATCGATTGATTGTGCATCATCTGATCGGACGTTT

Chainii TCAACCATGTGAGTACAATGACTGGGAATCGATTAATTGTGCATCATCTGATCGGGCGTTT

Floreyi TCAACCATGTGAGTACAATGACTGGGAATCGATTGATTGTGCATCATCTGATCGGGCGTTT

Reverse primer - not targeted

TCGACCAGAACGGCACG

Chrysogenum TCCATGGTACCGGGCTCCAAATCGACCAGAACGGCACGGGGAACGTACTTGTCACCGCTGG

Rubens TCCATGGTACCGGGCTCCAAATCGACCAGAACGGCACGGGGAACGTACTTGTCACCGCTGG

Chainii TCCATGGTACCGGGCTCCAAATCGACCAGAACGGCACGGGGAACGTACTTGTCACCGCTGG

Floreyi TCCATGGTACCGGGCTCCAAATCGACCAGAACGGCACGGGGAACGTACTTGTCACCGCTGG

***Penicillium rubens***

crt1_Rubens

Amplicon length - 180 bases

Forward primer - site 416, 425

CCTCGAGGACTTCAATCCTCCCGTG

Chrysogenum CAAGAAGGGCAGCCTCCTCGAGGACTTCAACCCTCCCGTCAACCCTGAGAAGGAGATTGAT

Rubens CAAGAAGGGCAGCCTCCTCGAGGACTTCAA**T**CCTCCCGT**G**AACCCTGAGAAGGAGATTGAT

Chainii CAAGAAGGGCAGCCTCCTCGAGGACTTCAACCCTCCCGTCAACCCTGAGAAGGAGATTGAT

Floreyi CAAGAAGGGCAGCCTCCTCGAGGACTTCAACCCTCCCGTCAACCCTGAGAAGGAGATTGAT

Reverse primer - site 563

GTCAGCGGGCTGAGTGGCC

Chrysogenum ---------------CCAGTCAGCGGGCTGAGTGGC**G**TCCTCATCGAGGATCTCGAAGGGA

Rubens ---------------CCAGTCAGCGGGCTGAGTGGCCTCCTCATCGAGGATCTCGAAGGGA

Chainii ---------------CCAGTCAGCGGGCTGAGTGGCCTCCTCATCGAGGATCTCGAAGGGA

Floreyi ---------------CCAGTCAGCGGGCTGAGTGGCCTCCTCATCGAGGATCTCGAAGGGA

***Penicillium chainii***

crt1_Chainii

Amplicon length - 185 bases

Forward primer - site 41, 64, 66, 69

CTTTCTACAATTGCTCGCGTTTTTATTTG

Chrysogenum CAGAGTGAGTCGTGACTTTCTACAATTGCTCGCGTTTTTATTTACTGACTGAAATTG

Rubens CAGAGTGAGTCGTGACTTTCTACAATTGCTCGCGTTTT**_**A**C**TTACTGACTGAAATTG

Chainii CAGAGTGAGTCGTGG**C**TTTCTACAATTGCTCGCGTTTTTATTT**G**CTGACTGAAATTG

Floreyi CAGAGTGAGTCGTGAATTTCTACAATTGCTCGCGTTTTTATTTACTGACTGAAATTG

Reverse primer - site 203

CCTTGTTAGTGGCACCGCACTTA

Chrysogenum TGGCGGAAGATGAAGTGAACCTTGTTAGTGGCACCGCACTTGTCAGGGCCGAACATG

Rubens TGGCGGAAGATGAAGTGAACCTTGTTAGTGGCACCGCACTTGTCAGGGCCGAACATG

Chainii TGGCGGAAGATGAAGTGAACCTTGTTAGTGGCACCGCACTT**A**TCAGGGCCGAACATG

Floreyi TGGCGGAAGATGAAATGAACCTTGTTAGTGGCACCGCACTTGTCAGGGCCGAACATG

***Penicillium floreyi***

parA_floreyi

Amplicon length - 111 bases

Forward primer - site 61, 71, 76

ACGGCCCCTCCTTACGAAA

Chrysogenum TTAATACCCATAACGACCCCTCCTTCCGAATCCCCTTTTATCTGACTCCTCCTTATAGAGA

Rubens TTAATACCCATAACGACCCCTCCTTCCGAAGCCCCTTTTATCTGACTCCTCCTTATAGAGA

Chainii TTAATACCCATAACGACCCCTCCTTCTGAAGCCCCTTTTATCTGACTCCTCCTTATAGAGA

Floreyi TTAATACCCATAACG**G**CCCCTCCTT**A**CGAA**A**CCCCTTTTATCTGACTCCTCCTTATAGAGA

Reverse primer - not targeted

TGTGAGACCAAAGGCAGTGG

Chrysogenum TGTCTAATGTAACAAGACCTGTGAGACCAAAGGCAGTGGTCTGGAAGAGATGAGGAA

Rubens TGTCTAATGTAACAAGACCTGTGAGACCAAAGGCAGTGGTCTGGAAGAGATGAGGAA

Chainii TGTCTAATGTAACAAGACCTGTGAGACCAAAGGCAGTGGTCTGGAAGAGATGAGGAA

Floreyi TGTCTAATGTAACAAGACCTGTGAGACCAAAGGCAGTGGTCTGGAAGAGATGAGGAA
